# Supplementary material for: Unlocking the Medicinal Mysteries: Preventing Lacunar Stroke with Drug Repurposing
Source: Biomedicines. 2023 Dec 20;12(1):17. doi: 10.3390/biomedicines12010017 (PMC10813761; doi:10.3390/biomedicines12010017)
Supplement: Supplementary file 1 [file biomedicines-12-00017-s001.zip › biomedicines-2702237-supplementary.pdf]

Table S1. all potential modifaiable risk factors classification groups.

modifiable risk factors	specific exposures
**anthropometry**	waist-to-hip ratio (WHR)
	Body fat
	Height
	Body Mass Index (BMI)
	Bone mineral density
	Childhood BMI
	Birth weight
**socioeconomic factors**	Education
	intelligence
**lifestyle/dietary factors**	diphenylamine (DPA)
	smoking (number)
	Eicosapntemacnioc Acid (EPA)
	Linoleic acid (LA; 18:2,n6)
	Coffee
	Morning person
	Subjective well-being
	Adrenic acid (22:4,n6)
	Arachidonic acid (AA; 20:4,n6)
	Sedentary
	Carbohydrate
	Gamma linolenic acid (GLA; 18:3,n6)
**cadiometabolic factors**	common carotid intima-media thickness
	Coronary heart disease
	HDL cholesterol || id:ieu-b-109
	Total cholesterol
	homocysteine (Hcy)
	C-reaction protein (CRP)
	Type 2 diabetes
	lipoprotein(a)
	Fasting glucose
	Heart rate
	Fasting proinsulin
	Fasting insulin
	Pulse pressure
	apolipoprotein A-I || id:ieu-b-107
	diastolic blood pressure (DBP)
	triglycerides || id:ieu-b-111
	Hypertension
	Fibrinogen
	Adiponectin
	Atrial fibrillation
	Leptin

	LDL cholesterol || id:ieu-b-110
	HbA1C
	2h glucose
	systolic blood pressure (SBP)
	apolipoprotein B || id:ieu-b-108
**endogenous substances**	Serum creatinine
	vitamin E
	Uric acid
	eGFRcrea
	Blood urea nitrogen
	Vitamin b12
	Protein
	vitamin D
**neuropsychiatric disorders**	anorexia nervosa
	Schizophrenia
	Neuroticism
	major depression
	Parkinson's disease
**other system diseases**	Chronic obstructive pulmonary disease
	Rheumatoid Arthritis
	Osteoporotic fracture
	Crohn's Disease
	Asthma

Table S2. Summarised GWAS data for potentially modifiable risk factors that shown significant effects on lacunar stroke in univariable MR.

Traits	No of Ivs in the study (P < 5 × 10-8)	Gwas	No of sample	Units
SBP	98	Nat Genet. 2018 Oct; 50(10): 1412–1425.	over one million people of European ancestry	mmHg
DBP	75			
PP	76			
HDL cholesterol || id:ieu-b-109	257	UK Biobank, Neale lab, https://gwas.mrcieu.ac.uk/	403,943	mmol/L
apolipoprotein A-I || id:ieu-b-107	226		393,193	mmol/L
triglycerides || id:ieu-b-111	234		441,016	mmol/L
apolipoprotein B || id:ieu-b-108	142		439,214	mmol/L
Type 2 diabetes	113	Nature Com. 2018;9(1):2941.	659,316	increased t2d risk (OR)
Height	290	Nat Genet. 2014 Nov; 46(11): 1173–1186.	253,288	m
Education	30	Nature. 2016 May 26;533(7604):539-42.	293,723	years of schooling
Fasting proinsulin	9	Diabetes 2011;60(10):2624–2634	27,079	pmol/L
Fibrinogen	33	Hum Mol Genet. 2016 Jan 15; 25(2): 358–370.	120,246	g/l
Atrial fibrillation	90	Nat Genet. 2018 Sep; 50(9): 1225–1233.	over half a million individuals including 65,446 with AF	increased AF risk (OR)

Table S3. Genetic variants included in drug-target analyses for each region.

Traits	rsid	a1	a2	a1_freq	effect	SE	p-value
Adrenergic neurone blockers-DBP	rs2692938	A	G	0.7879	-0.0212	0.003346	2.36E-10
Adrenergic neurone blockers-DBP	rs3111873	C	G	0.6675	0.02543	0.002903	1.948E-18
Adrenergic neurone blockers-PP	rs10171471	C	T	0.7366	0.0283	0.003599	3.763E-15
Adrenergic neurone blockers-PP	rs3111873	C	G	0.6675	-0.02408	0.003342	5.803E-13
Adrenergic neurone blockers-PP	rs4526784	C	G	0.3742	0.02026	0.003236	3.832E-10
Adrenergic neurone blockers-SBP	rs4526784	C	G	0.3742	0.02826	0.004899	8.022E-09
Alpha-adrenoceptor blockers-DBP	rs1741288	A	G	0.6384	-0.01833	0.002847	1.201E-10
Alpha-adrenoceptor blockers-DBP	rs217728	T	C	0.253	-0.02238	0.00314	1.018E-12
Alpha-adrenoceptor blockers-DBP	rs2735461	G	C	0.9452	-0.03409	0.005994	1.291E-08
Alpha-adrenoceptor blockers-DBP	rs415196	T	C	0.2749	0.01949	0.003072	2.224E-10
Alpha-adrenoceptor blockers-DBP	rs45471201	T	C	0.09837	-0.02621	0.00461	1.308E-08
Alpha-adrenoceptor blockers-DBP	rs4872453	C	T	0.2532	-0.01901	0.003128	1.221E-09
Alpha-adrenoceptor blockers-PP	rs141501760	T	G	0.02859	-0.06011	0.009888	1.213E-09
Alpha-adrenoceptor blockers-PP	rs217728	T	C	0.253	-0.03471	0.003609	6.835E-22
Alpha-adrenoceptor blockers-SBP	rs112492	G	A	0.2578	0.03341	0.005404	6.329E-10
Alpha-adrenoceptor blockers-SBP	rs141501760	T	G	0.02859	-0.1035	0.014939	4.271E-12
Alpha-adrenoceptor blockers-SBP	rs217728	T	C	0.253	-0.05864	0.005468	7.879E-27
Alpha-adrenoceptor blockers-SBP	rs2735461	G	C	0.9452	-0.06705	0.010452	1.405E-10
Angiotensin converting enzyme inhibitors-DBP	rs4968783	A	C	0.6179	0.01895	0.002808	1.494E-11
Angiotensin converting enzyme inhibitors-SBP	rs4968783	A	C	0.6179	0.02998	0.004891	8.84E-10
Angiotensin-II receptor antagonists-PP	rs71304101	A	G	0.1181	0.03005	0.004854	6.011E-10
Beta-adrenoceptor blockers-DBP	rs11196589	C	A	0.3982	-0.01869	0.002815	3.127E-11
Beta-adrenoceptor blockers-DBP	rs117624845	A	G	0.07281	-0.04545	0.005254	5.117E-18
Beta-adrenoceptor blockers-DBP	rs13166730	T	C	0.17	-0.02105	0.00375	1.984E-08
Beta-adrenoceptor blockers-DBP	rs13242223	G	A	0.2339	-0.01808	0.003246	2.548E-08
Beta-adrenoceptor blockers-DBP	rs151545	A	C	0.1256	0.02421	0.00428	1.545E-08
Beta-adrenoceptor blockers-DBP	rs1741288	A	G	0.6384	-0.01833	0.002847	1.201E-10
Beta-adrenoceptor blockers-DBP	rs17653278	G	C	0.05661	0.03425	0.005883	5.825E-09
Beta-adrenoceptor blockers-DBP	rs180940	G	A	0.6693	-0.02813	0.002904	3.448E-22
Beta-adrenoceptor blockers-DBP	rs2782980	C	T	0.7209	-0.03911	0.003033	4.961E-38
Beta-adrenoceptor blockers-DBP	rs2888691	A	G	0.1496	0.0235	0.00384	9.346E-10
Beta-adrenoceptor blockers-DBP	rs2907947	G	A	0.6218	0.0198	0.002829	2.563E-12
Beta-adrenoceptor blockers-DBP	rs35320559	C	T	0.2248	0.0211	0.003274	1.161E-10
Beta-adrenoceptor blockers-DBP	rs3918226	T	C	0.07838	-0.08164	0.005151	1.49E-56
Beta-adrenoceptor blockers-DBP	rs41313071	A	C	0.04352	0.04068	0.006739	1.583E-09
Beta-adrenoceptor blockers-DBP	rs415196	T	C	0.2749	0.01949	0.003072	2.224E-10
Beta-adrenoceptor blockers-DBP	rs45471201	T	C	0.09837	-0.02621	0.00461	1.308E-08
Beta-adrenoceptor blockers-DBP	rs4872453	C	T	0.2532	-0.01901	0.003128	1.221E-09

Beta-adrenoceptor blockers-DBP	rs68122733	G	A	0.1707	0.02935	0.00363	6.195E-16
Beta-adrenoceptor blockers-DBP	rs72829191	T	C	0.09773	-0.02771	0.004652	2.58E-09
Beta-adrenoceptor blockers-DBP	rs740746	A	G	0.7326	-0.03846	0.003084	1.092E-35
Beta-adrenoceptor blockers-DBP	rs740956	C	T	0.4364	0.02149	0.002739	4.35E-15
Beta-adrenoceptor blockers-DBP	rs741066	T	C	0.2942	-0.01895	0.002984	2.15E-10
Beta-adrenoceptor blockers-DBP	rs74157560	T	C	0.04578	-0.04864	0.006553	1.15E-13
Beta-adrenoceptor blockers-DBP	rs75228369	T	C	0.04795	-0.04062	0.006536	5.152E-10
Beta-adrenoceptor blockers-DBP	rs753482	A	C	0.7915	0.0249	0.003387	1.956E-13
Beta-adrenoceptor blockers-DBP	rs77021631	G	A	0.1831	0.02924	0.003519	9.571E-17
Beta-adrenoceptor blockers-DBP	rs7737361	A	G	0.2028	0.02136	0.003474	7.788E-10
Beta-adrenoceptor blockers-DBP	rs79043825	A	C	0.03583	-0.04211	0.007345	9.861E-09
Beta-adrenoceptor blockers-DBP	rs855715	T	G	0.1177	0.04714	0.004302	6.104E-28
Beta-adrenoceptor blockers-DBP	rs891511	A	G	0.3279	0.03806	0.002942	2.771E-38
Beta-adrenoceptor blockers-DBP	rs917875	C	A	0.04807	0.04424	0.006453	7.098E-12
Beta-adrenoceptor blockers-PP	rs74157560	T	C	0.04578	-0.04333	0.007505	7.772E-09
Beta-adrenoceptor blockers-SBP	rs117624845	A	G	0.07281	-0.06348	0.009161	4.24E-12
Beta-adrenoceptor blockers-SBP	rs13166730	T	C	0.17	-0.04141	0.006541	2.439E-10
Beta-adrenoceptor blockers-SBP	rs180940	G	A	0.6693	-0.04251	0.005057	4.244E-17
Beta-adrenoceptor blockers-SBP	rs2782980	C	T	0.7209	-0.05801	0.005283	4.773E-28
Beta-adrenoceptor blockers-SBP	rs35320559	C	T	0.2248	0.03729	0.005705	6.297E-11
Beta-adrenoceptor blockers-SBP	rs3918226	T	C	0.07838	-0.09769	0.00899	1.679E-27
Beta-adrenoceptor blockers-SBP	rs68122733	G	A	0.1707	0.04358	0.006329	5.759E-12
Beta-adrenoceptor blockers-SBP	rs740746	A	G	0.7326	-0.05422	0.005371	5.809E-24
Beta-adrenoceptor blockers-SBP	rs740956	C	T	0.4364	0.03184	0.004772	2.519E-11
Beta-adrenoceptor blockers-SBP	rs741066	T	C	0.2942	-0.02859	0.005201	3.872E-08
Beta-adrenoceptor blockers-SBP	rs74157560	T	C	0.04578	-0.0936	0.011392	2.103E-16
Beta-adrenoceptor blockers-SBP	rs753482	A	C	0.7915	0.03296	0.005901	2.325E-08
Beta-adrenoceptor blockers-SBP	rs77021631	G	A	0.1831	0.04808	0.006132	4.482E-15
Beta-adrenoceptor blockers-SBP	rs855715	T	G	0.1177	0.06657	0.007504	7.232E-19
Beta-adrenoceptor blockers-SBP	rs891511	A	G	0.3279	0.05557	0.005123	2.061E-27
Calcium channel blockers-DBP	rs10764331	G	A	0.418	-0.02625	0.002762	2.061E-21
Calcium channel blockers-DBP	rs10828452	T	A	0.2049	0.01897	0.00345	3.818E-08
Calcium channel blockers-DBP	rs10828749	A	G	0.4104	0.02259	0.002793	6.088E-16
Calcium channel blockers-DBP	rs11062219	T	C	0.3678	-0.02065	0.002835	3.235E-13
Calcium channel blockers-DBP	rs113210396	T	G	0.04499	0.03838	0.006585	5.604E-09
Calcium channel blockers-DBP	rs11591541	G	A	0.1571	-0.02516	0.003751	1.978E-11
Calcium channel blockers-DBP	rs11720002	C	T	0.2723	-0.01922	0.003055	3.149E-10
Calcium channel blockers-DBP	rs12258967	G	C	0.295	0.04153	0.002987	6.092E-44
Calcium channel blockers-DBP	rs1277754	G	A	0.7107	0.0182	0.003034	1.992E-09
Calcium channel blockers-DBP	rs1325990	G	A	0.5367	-0.02532	0.00274	2.486E-20
Calcium channel blockers-DBP	rs16916944	T	C	0.1361	-0.02668	0.004024	3.34E-11
Calcium channel blockers-DBP	rs3774751	T	G	0.4617	0.01918	0.002735	2.351E-12
Calcium channel blockers-DBP	rs3819531	T	C	0.7033	-0.01745	0.002971	4.29E-09
Calcium channel blockers-DBP	rs3821843	A	G	0.6796	-0.0255	0.002966	8.189E-18

Calcium channel blockers-DBP	rs4748478	A	G	0.3762	-0.01853	0.002807	4.098E-11
Calcium channel blockers-DBP	rs61278674	G	A	0.0988	-0.02709	0.004696	8.005E-09
Calcium channel blockers-DBP	rs67214975	A	C	0.454	0.02727	0.002742	2.666E-23
Calcium channel blockers-DBP	rs7314860	A	G	0.1733	0.0218	0.003654	2.434E-09
Calcium channel blockers-DBP	rs7340705	C	T	0.3268	-0.02176	0.002931	1.13E-13
Calcium channel blockers-DBP	rs76719841	C	T	0.0376	-0.04039	0.007201	2.036E-08
Calcium channel blockers-PP	rs10764331	G	A	0.418	-0.02422	0.003175	2.398E-14
Calcium channel blockers-PP	rs10828749	A	G	0.4104	0.01887	0.003212	4.215E-09
Calcium channel blockers-PP	rs11248862	G	A	0.8731	0.02725	0.004753	9.88E-09
Calcium channel blockers-PP	rs11591541	G	A	0.1571	-0.03572	0.004317	1.286E-16
Calcium channel blockers-PP	rs12258967	G	C	0.295	0.03851	0.003437	3.939E-29
Calcium channel blockers-PP	rs1325990	G	A	0.5367	-0.02449	0.003153	8.036E-15
Calcium channel blockers-PP	rs13429172	C	A	0.3908	0.01778	0.003224	3.484E-08
Calcium channel blockers-PP	rs144399820	C	T	0.1404	-0.02991	0.00456	5.434E-11
Calcium channel blockers-PP	rs150857355	C	G	0.0217	-0.06581	0.010992	2.139E-09
Calcium channel blockers-PP	rs1779246	A	G	0.8005	0.03021	0.003956	2.226E-14
Calcium channel blockers-PP	rs2497818	G	A	0.1066	-0.03132	0.005075	6.75E-10
Calcium channel blockers-PP	rs3737984	T	G	0.4094	-0.01829	0.003189	9.76E-09
Calcium channel blockers-PP	rs3821843	A	G	0.6796	-0.02322	0.003414	1.044E-11
Calcium channel blockers-PP	rs4748472	T	C	0.6621	-0.01831	0.003347	4.477E-08
Calcium channel blockers-PP	rs508011	A	G	0.3693	0.02025	0.003274	6.224E-10
Calcium channel blockers-PP	rs67214975	A	C	0.454	0.02678	0.003156	2.138E-17
Calcium channel blockers-PP	rs6792713	T	G	0.01564	-0.07387	0.01241	2.635E-09
Calcium channel blockers-PP	rs71384617	T	C	0.3557	0.01863	0.003386	3.773E-08
Calcium channel blockers-PP	rs7894090	A	G	0.8551	0.02856	0.004464	1.583E-10
Calcium channel blockers-PP	rs7902194	T	A	0.7389	0.02424	0.003615	2.023E-11
Calcium channel blockers-PP	rs7920075	T	C	0.5289	0.018	0.003166	1.305E-08
Calcium channel blockers-SBP	rs10764331	G	A	0.418	-0.05149	0.00481	9.835E-27
Calcium channel blockers-SBP	rs10828452	T	A	0.2049	0.03633	0.006013	1.52E-09
Calcium channel blockers-SBP	rs10828749	A	G	0.4104	0.04209	0.004865	5.097E-18
Calcium channel blockers-SBP	rs11248862	G	A	0.8731	0.03994	0.007188	2.749E-08
Calcium channel blockers-SBP	rs113210396	T	G	0.04499	0.06767	0.011487	3.84E-09
Calcium channel blockers-SBP	rs11591541	G	A	0.1571	-0.06238	0.006535	1.363E-21
Calcium channel blockers-SBP	rs116936375	A	G	0.03662	0.07097	0.012751	2.606E-08
Calcium channel blockers-SBP	rs12258967	G	C	0.295	0.08164	0.005204	1.873E-55
Calcium channel blockers-SBP	rs1277754	G	A	0.7107	0.03274	0.005285	5.822E-10
Calcium channel blockers-SBP	rs1325990	G	A	0.5367	-0.05094	0.004773	1.389E-26
Calcium channel blockers-SBP	rs150857355	C	G	0.0217	-0.1167	0.016625	2.233E-12
Calcium channel blockers-SBP	rs16916944	T	C	0.1361	-0.04939	0.007013	1.887E-12
Calcium channel blockers-SBP	rs17123349	G	A	0.09325	-0.04862	0.008158	2.526E-09
Calcium channel blockers-SBP	rs1779246	A	G	0.8005	0.04925	0.005989	1.978E-16
Calcium channel blockers-SBP	rs2497818	G	A	0.1066	-0.05262	0.007695	8.012E-12
Calcium channel blockers-SBP	rs3819531	T	C	0.7033	-0.02885	0.005177	2.508E-08
Calcium channel blockers-SBP	rs3821843	A	G	0.6796	-0.0499	0.005168	4.709E-22

Calcium channel blockers-SBP	rs4748472	T	C	0.6621	-0.0336	0.005064	3.239E-11
Calcium channel blockers-SBP	rs61278674	G	A	0.0988	-0.0472	0.008178	7.864E-09
Calcium channel blockers-SBP	rs67214975	A	C	0.454	0.05505	0.004777	1.013E-30
Calcium channel blockers-SBP	rs72957281	C	T	0.231	-0.03309	0.005625	4.05E-09
Calcium channel blockers-SBP	rs7340705	C	T	0.3268	-0.03579	0.005107	2.406E-12
Calcium channel blockers-SBP	rs76719841	C	T	0.0376	-0.08408	0.012556	2.141E-11
Calcium channel blockers-SBP	rs7894090	A	G	0.8551	0.04157	0.006758	7.691E-10
Calcium channel blockers-SBP	rs7920075	T	C	0.5289	0.02926	0.004793	1.027E-09
Centrally acting antihypertensives-DBP	rs2692938	A	G	0.7879	-0.0212	0.003346	2.36E-10
Centrally acting antihypertensives-DBP	rs3111873	C	G	0.6675	0.02543	0.002903	1.948E-18
Centrally acting antihypertensives-PP	rs10171471	C	T	0.7366	0.0283	0.003599	3.763E-15
Centrally acting antihypertensives-PP	rs11718509	A	G	0.3807	-0.02143	0.003234	3.44E-11
Centrally acting antihypertensives-PP	rs3111873	C	G	0.6675	-0.02408	0.003342	5.803E-13
Loop diuretics-DBP	rs331077	T	C	0.425	-0.02093	0.002767	3.886E-14
Loop diuretics-DBP	rs7718312	C	T	0.2845	0.02122	0.003018	2.059E-12
Loop diuretics-MAP	rs331077	T	C	0.425	-0.02516	0.003265	1.292E-14
Loop diuretics-MAP	rs7718312	C	T	0.2845	0.02755	0.003561	1.033E-14
Loop diuretics-PP	rs2015637	C	T	0.1009	0.06118	0.005184	3.868E-32
Loop diuretics-PP	rs35026266	T	C	0.2482	0.0239	0.003628	4.504E-11
Loop diuretics-SBP	rs2015637	C	T	0.1009	0.04711	0.007862	2.068E-09
Loop diuretics-SBP	rs331077	T	C	0.425	-0.03282	0.004822	1.003E-11
Loop diuretics-SBP	rs7718312	C	T	0.2845	0.03923	0.00526	8.804E-14
PSDs and aldosterone antagonists-PP	rs1058161	T	C	0.02403	0.06098	0.010302	3.237E-09
PSDs and aldosterone antagonists-PP	rs2649599	G	A	0.7894	-0.0281	0.003948	1.096E-12
PSDs and aldosterone antagonists-PP	rs307349	T	C	0.9222	-0.04558	0.005923	1.42E-14
PSDs and aldosterone antagonists-PP	rs35975487	G	A	0.05493	-0.03947	0.006838	7.832E-09
PSDs and aldosterone antagonists-SBP	rs307349	T	C	0.9222	-0.05641	0.00902	4.014E-10
Renin inhibitors-DBP	rs11240656	A	G	0.4633	-0.01534	0.002742	2.223E-08
Renin inhibitors-DBP	rs16852778	T	C	0.1622	-0.02162	0.003713	5.785E-09
Renin inhibitors-DBP	rs56305552	A	G	0.502	0.01499	0.002724	3.738E-08
Renin inhibitors-SBP	rs11240656	A	G	0.4633	-0.03151	0.004776	4.188E-11
Renin inhibitors-SBP	rs4293010	T	G	0.9235	-0.0544	0.008956	1.251E-09
Thiazides and related diuretics-PP	rs12141314	G	A	0.1695	-0.02576	0.004166	6.262E-10
Thiazides and related diuretics-PP	rs2015637	C	T	0.1009	0.06118	0.005184	3.868E-32
Thiazides and related diuretics-PP	rs2474453	T	G	0.4938	0.02482	0.003194	7.778E-15
Thiazides and related diuretics-PP	rs35026266	T	C	0.2482	0.0239	0.003628	4.504E-11
Thiazides and related diuretics-PP	rs72634852	T	C	0.08407	0.03435	0.005672	1.396E-09
Thiazides and related diuretics-SBP	rs12141314	G	A	0.1695	-0.03636	0.00631	8.311E-09
Thiazides and related diuretics-SBP	rs2015637	C	T	0.1009	0.04711	0.007862	2.068E-09
Thiazides and related diuretics-SBP	rs2474453	T	G	0.4938	0.03381	0.004832	2.631E-12
Thiazides and related diuretics-SBP	rs3128290	T	G	0.6035	-0.02723	0.004857	2.07E-08
Vasodilator antihypertensives-DBP	rs41464847	G	A	0.4798	-0.02017	0.002723	1.3E-13
Vasodilator antihypertensives-PP	rs11024256	G	T	0.345	0.02403	0.00331	3.855E-13
Vasodilator antihypertensives-PP	rs214085	T	C	0.4113	-0.02765	0.003186	4.045E-18

Vasodilator antihypertensives-PP	rs4526784	C	G	0.3742	0.02026	0.003236	3.832E-10
Vasodilator antihypertensives-PP	rs56228409	C	A	0.1541	-0.02669	0.004435	1.756E-09
Vasodilator antihypertensives-PP	rs61755606	A	G	0.1079	0.03806	0.005106	9.026E-14
Vasodilator antihypertensives-PP	rs6855875	T	C	0.1906	-0.02854	0.004002	9.952E-13
Vasodilator antihypertensives-PP	rs7928810	A	C	0.6238	0.03342	0.003248	7.92E-25
Vasodilator antihypertensives-SBP	rs11024256	G	T	0.345	0.03557	0.005011	1.262E-12
Vasodilator antihypertensives-SBP	rs214085	T	C	0.4113	-0.03797	0.004827	3.665E-15
Vasodilator antihypertensives-SBP	rs4526784	C	G	0.3742	0.02826	0.004899	8.022E-09
Vasodilator antihypertensives-SBP	rs7928810	A	C	0.6238	0.04449	0.004914	1.387E-19
**LDL-lowering target weighted by LDL**							
APOC3	rs10790162	A	G	0.1	0.2305	0.0065	1.00E-200
APOC3	rs603446	C	T	0.55	0.0502	0.0034	3.91E-43
NPC1L1	rs10234070	T	C	0.09631	0.0295	0.0059	0.00000152
NPC1L1	rs217386	G	A	0.5923	0.0363	0.0038	1.20E-19
NPC1L1	rs2300414	A	G	0.06992	0.0353	0.008	0.00000545
NPC1L1	rs7791240	C	T	0.09103	0.0425	0.0065	1.84E-10
NPC1L1	rs2073547	G	A	0.195	0.0485	0.0049	1.92E-21
HMGCR	rs12916	C	T	0.4314	0.0733	0.0038	7.79E-78
HMGCR	rs17238484	T	G	0.2533	0.0627	0.0062	1.35E-21
HMGCR	rs2006760	G	C	0.229	0.0533	0.0076	1.67E-13
HMGCR	rs2303152	A	G	0.1201	0.0423	0.0064	1.04E-09
HMGCR	rs5909	A	G	0.1016	0.0617	0.0088	4.93E-13
HMGCR	rs10066707	A	G	0.396	0.05	0.005	3.00E-19
PCSK9	rs10493176	T	G	0.885	0.078	0.01	2.50E-14
PCSK9	rs11206510	T	C	0.846	0.083	0.005	2.38E-53
PCSK9	rs11206514	A	C	0.611	0.051	0.004	1.00E-32
PCSK9	rs11583974	A	G	0.03	0.065	0.012	0.000000004
PCSK9	rs11591147	G	T	0.983	0.497	0.018	8.60E-143
PCSK9	rs12067569	A	G	0.034	0.089	0.01	2.00E-17
PCSK9	rs2479394	G	A	0.285	0.039	0.004	1.60E-19
PCSK9	rs2479409	G	A	0.333	0.064	0.004	2.51E-50
PCSK9	rs2495477	T	C	0.6	0.064	0.005	7.30E-30
PCSK9	rs572512	T	C	0.346	0.048	0.005	5.30E-26
PCSK9	rs585131	T	C	0.815	0.064	0.005	2.70E-35
**triglyceride-lowering target weighted by triglyceride**							
ANGPTL3	rs4587594	G	A	0.69	0.069	0.004	3.50E-82
APOB	rs676210	G	A	0.769	0.073	0.004	3.28E-71
APOA5/APOC3	rs12280753	T	C	6.70E-02	0.193	0.006	1.22E-179
APOA5/APOC3	rs7350481	T	C	0.098	0.225	0.007	1.00E-200
LPL	rs12678919	A	G	0.879	0.17	0.006	1.82E-199
**antidiabetic drug target**							

GLP1R	rs10305420	T	C	0.39	-0.051	0.016	1.30x10-3
GLP1R	rs75151020	C	A	0.09	0.119	0.026	7.08x10-6
GLP1R	rs2268647	T	C	0.52	0.066	0.015	1.51x10-5
**general glycemic control**							
glycemic control	rs7554251	C	T	0.73	0.036	0.017	3.39E-02
glycemic control	rs1127215	T	C	0.42	-0.065	0.016	5.67E-05
glycemic control	rs66464442	A	C	0.32	0.121	0.016	2.05E-13
glycemic control	rs1493694	T	C	0.11	0.146	0.025	1.05E-08
glycemic control	rs145904381	C	T	0.01	-0.266	0.071	1.98E-04
glycemic control	rs2297607	G	A	0.24	0.051	0.018	4.65E-03
glycemic control	rs6696888	A	G	0.68	-0.048	0.016	2.75E-03
glycemic control	rs7546252	G	A	0.56	-0.092	0.015	1.97E-09
glycemic control	rs539515	C	A	0.21	0.049	0.019	9.89E-03
glycemic control	rs2816177	G	A	0.41	0.049	0.016	2.25E-03
glycemic control	rs41304257	G	A	0.28	-0.042	0.017	1.34E-02
glycemic control	rs61817176	C	A	0.52	-0.074	0.015	1.17E-06
glycemic control	rs10916780	G	A	0.2	-0.045	0.019	1.77E-02
glycemic control	rs340874	C	T	0.57	0.166	0.015	2.68E-26
glycemic control	rs1337101	T	G	0.32	-0.095	0.016	6.05E-09
glycemic control	rs348330	A	G	0.63	-0.119	0.016	4.90E-13
glycemic control	rs10925635	C	A	0.64	0.046	0.016	4.09E-03
glycemic control	rs17261915	C	T	0.25	0.074	0.018	4.64E-05
glycemic control	rs3753693	T	C	0.41	-0.066	0.016	4.38E-05
glycemic control	rs61779284	A	G	0.21	0.13	0.019	2.58E-11
glycemic control	rs79090772	C	T	0.09	-0.219	0.027	3.87E-15
glycemic control	rs2269247	T	C	0.18	-0.056	0.02	5.15E-03
glycemic control	rs11583755	C	A	0.36	0.107	0.016	6.88E-11
glycemic control	rs2613499	G	A	0.19	-0.052	0.019	6.23E-03
glycemic control	rs10159026	T	C	0.25	-0.062	0.018	6.08E-04
glycemic control	rs2482506	G	C	0.25	-0.053	0.018	3.29E-03
glycemic control	rs11196174	G	A	0.29	0.252	0.017	4.84E-45
glycemic control	rs149692182	T	C	0.02	0.309	0.053	1.11E-08
glycemic control	rs35676242	A	C	0.05	0.23	0.036	4.33E-10
glycemic control	rs11257655	T	C	0.21	0.219	0.019	2.56E-28
glycemic control	rs946859	A	G	0.47	-0.075	0.015	8.44E-07
glycemic control	rs3122231	C	T	0.65	0.05	0.016	1.83E-03
glycemic control	rs113899647	T	C	0.03	-0.189	0.044	2.13E-05
glycemic control	rs949693	A	G	0.61	-0.05	0.016	1.83E-03
glycemic control	rs11592899	A	G	0.34	-0.055	0.016	6.23E-04
glycemic control	rs2812535	A	G	0.62	0.069	0.016	1.98E-05
glycemic control	rs697239	C	T	0.46	-0.105	0.015	9.28E-12
glycemic control	rs11201992	A	C	0.46	-0.038	0.015	1.13E-02
glycemic control	rs1111875	T	C	0.41	-0.181	0.016	2.28E-27
glycemic control	rs66536955	C	T	0.26	0.044	0.017	9.63E-03

glycemic control	rs34041345	G	T	0.26	0.06	0.018	9.01E-04
glycemic control	rs529623	C	T	0.52	-0.059	0.015	9.55E-05
glycemic control	rs10893830	T	C	0.13	-0.058	0.023	1.16E-02
glycemic control	rs10750397	G	A	0.72	-0.04	0.017	1.85E-02
glycemic control	rs67232546	T	C	0.21	0.067	0.019	4.52E-04
glycemic control	rs117316450	G	C	0.02	0.316	0.054	9.80E-09
glycemic control	rs757110	A	C	0.64	-0.112	0.016	9.28E-12
glycemic control	rs11042987	A	C	0.58	-0.034	0.016	3.33E-02
glycemic control	rs10831668	T	C	0.02	0.234	0.06	1.09E-04
glycemic control	rs231362	G	A	0.52	0.12	0.015	8.84E-15
glycemic control	rs10767659	T	G	0.67	-0.041	0.016	1.04E-02
glycemic control	rs60808706	A	G	0.05	-0.227	0.035	2.40E-10
glycemic control	rs2289488	C	G	0.4	0.04	0.016	1.24E-02
glycemic control	rs62618693	T	C	0.05	-0.144	0.037	1.13E-04
glycemic control	rs523472	A	G	0.72	-0.056	0.017	1.03E-03
glycemic control	rs7483027	C	T	0.38	-0.061	0.016	1.54E-04
glycemic control	rs174541	C	T	0.36	-0.098	0.016	2.07E-09
glycemic control	rs1143756	G	A	0.29	0.1	0.017	8.26E-09
glycemic control	rs3918296	G	C	0.03	-0.249	0.049	5.65E-07
glycemic control	rs11602873	T	A	0.16	-0.187	0.021	7.97E-18
glycemic control	rs4945090	A	T	0.6	0.036	0.016	2.43E-02
glycemic control	rs12802861	T	C	0.28	-0.052	0.017	2.28E-03
glycemic control	rs10830963	G	C	0.28	0.297	0.017	2.61E-61
glycemic control	rs3020069	A	G	0.68	0.093	0.016	1.22E-08
glycemic control	rs1426371	A	G	0.26	-0.074	0.018	4.64E-05
glycemic control	rs79310463	T	C	0.13	0.104	0.023	7.91E-06
glycemic control	rs56348580	C	G	0.31	-0.037	0.017	2.93E-02
glycemic control	rs7975763	T	C	0.2	-0.057	0.019	2.75E-03
glycemic control	rs11614914	T	C	0.33	0.078	0.016	1.54E-06
glycemic control	rs10841886	C	T	0.23	-0.082	0.018	6.79E-06
glycemic control	rs1480029	A	G	0.46	0.042	0.015	5.15E-03
glycemic control	rs3751239	G	C	0.2	-0.16	0.019	3.68E-16
glycemic control	rs11063018	C	T	0.17	0.067	0.02	8.50E-04
glycemic control	rs74862545	T	C	0.02	-0.279	0.052	1.34E-07
glycemic control	rs2732469	A	T	0.43	-0.258	0.015	1.57E-59
glycemic control	rs61937817	G	T	0.11	0.06	0.024	1.24E-02
glycemic control	rs11173646	T	A	0.82	-0.046	0.02	2.13E-02
glycemic control	rs2257883	A	G	0.13	0.15	0.023	1.93E-10
glycemic control	rs12371967	C	T	0.17	-0.043	0.02	3.13E-02
glycemic control	rs10879261	G	T	0.41	0.068	0.016	2.59E-05
glycemic control	rs11108094	A	C	0.07	0.099	0.03	1.01E-03
glycemic control	rs6538805	C	T	0.39	-0.076	0.016	2.78E-06
glycemic control	rs9587811	A	C	0.41	-0.056	0.016	4.98E-04
glycemic control	rs314879	T	C	0.79	-0.069	0.019	3.07E-04

glycemic control	rs34584161	G	A	0.24	-0.063	0.018	4.98E-04
glycemic control	rs380854	A	G	0.58	-0.058	0.016	3.14E-04
glycemic control	rs9316500	G	T	0.29	-0.067	0.017	9.26E-05
glycemic control	rs7991679	A	T	0.16	-0.081	0.021	1.29E-04
glycemic control	rs1215451	A	G	0.29	-0.131	0.017	7.45E-14
glycemic control	rs2295388	A	G	0.22	-0.073	0.019	1.37E-04
glycemic control	rs4906272	T	C	0.16	0.046	0.021	2.83E-02
glycemic control	rs12883788	T	C	0.46	0.06	0.015	7.31E-05
glycemic control	rs7147483	C	T	0.25	-0.158	0.018	2.22E-17
glycemic control	rs723355	A	G	0.5	-0.034	0.015	2.32E-02
glycemic control	rs4902002	A	G	0.71	-0.034	0.017	4.51E-02
glycemic control	rs242105	C	A	0.28	0.062	0.017	2.89E-04
glycemic control	rs7156625	A	G	0.22	0.037	0.019	5.11E-02
glycemic control	rs8010382	G	A	0.41	0.046	0.016	4.09E-03
glycemic control	rs8043085	T	G	0.23	0.071	0.018	9.14E-05
glycemic control	rs11856877	G	A	0.11	0.071	0.024	3.15E-03
glycemic control	rs1473781	A	G	0.35	0.067	0.016	3.37E-05
glycemic control	rs149336329	T	G	0.05	-0.272	0.037	8.86E-13
glycemic control	rs7163757	T	C	0.43	-0.042	0.015	5.15E-03
glycemic control	rs7178762	T	C	0.55	-0.057	0.015	1.61E-04
glycemic control	rs9479	G	A	0.49	0.052	0.015	5.61E-04
glycemic control	rs8033589	A	G	0.76	0.058	0.018	1.32E-03
glycemic control	rs12910361	G	A	0.71	0.161	0.017	7.02E-20
glycemic control	rs893617	T	C	0.72	-0.136	0.017	8.84E-15
glycemic control	rs2290202	T	G	0.13	0.085	0.023	2.41E-04
glycemic control	rs9927842	C	T	0.84	-0.056	0.021	7.67E-03
glycemic control	rs8056890	A	G	0.36	0.105	0.016	1.50E-10
glycemic control	rs8054556	A	G	0.47	0.077	0.015	4.37E-07
glycemic control	rs55857387	C	T	0.2	-0.142	0.019	3.81E-13
glycemic control	rs8061528	T	C	0.21	0.092	0.019	1.80E-06
glycemic control	rs2024449	C	T	0.44	-0.056	0.015	2.09E-04
glycemic control	rs1421085	C	T	0.4	0.154	0.016	1.84E-20
glycemic control	rs56125990	G	A	0.15	0.065	0.021	2.02E-03
glycemic control	rs4788815	T	A	0.66	0.056	0.016	4.98E-04
glycemic control	rs72802365	C	G	0.08	-0.163	0.029	3.48E-08
glycemic control	rs2966117	T	G	0.48	0.059	0.015	9.55E-05
glycemic control	rs11117364	G	A	0.68	0.066	0.017	1.17E-04
glycemic control	rs66461358	C	T	0.15	0.06	0.021	4.32E-03
glycemic control	rs12934854	A	G	0.17	0.043	0.02	3.13E-02
glycemic control	rs925095	T	C	0.39	-0.075	0.016	3.72E-06
glycemic control	rs2297508	G	C	0.65	-0.15	0.016	1.59E-19
glycemic control	rs9913225	A	G	0.58	-0.075	0.016	3.72E-06
glycemic control	rs1109442	C	T	0.47	0.071	0.015	3.01E-06
glycemic control	rs3110641	G	A	0.78	0.091	0.019	2.31E-06

glycemic control	rs11651755	T	C	0.51	-0.124	0.015	1.20E-15
glycemic control	rs3786017	C	T	0.11	0.054	0.025	3.05E-02
glycemic control	rs8071043	C	T	0.33	0.066	0.016	4.38E-05
glycemic control	rs1905339	C	T	0.34	0.097	0.016	2.96E-09
glycemic control	rs35895680	A	C	0.33	-0.089	0.016	4.76E-08
glycemic control	rs366577	T	C	0.6	-0.051	0.016	1.49E-03
glycemic control	rs57767539	A	G	0.07	0.136	0.031	1.43E-05
glycemic control	rs11658220	A	G	0.1	0.1	0.025	7.31E-05
glycemic control	rs12603589	C	T	0.19	0.103	0.02	4.02E-07
glycemic control	rs7224711	T	C	0.53	-0.069	0.015	5.56E-06
glycemic control	rs303760	T	C	0.35	0.052	0.016	1.20E-03
glycemic control	rs16965062	T	C	0.43	0.034	0.015	2.32E-02
glycemic control	rs7227272	A	G	0.1	-0.062	0.026	1.70E-02
glycemic control	rs410150	T	C	0.8	-0.047	0.019	1.33E-02
glycemic control	rs17596995	A	G	0.2	-0.049	0.019	9.89E-03
glycemic control	rs1517037	T	C	0.19	-0.093	0.02	4.42E-06
glycemic control	rs6567160	C	T	0.23	0.097	0.018	1.19E-07
glycemic control	rs74625348	C	G	0.23	-0.044	0.019	2.04E-02
glycemic control	rs12963820	A	T	0.27	0.034	0.017	4.51E-02
glycemic control	rs7240767	C	T	0.39	0.063	0.016	9.39E-05
glycemic control	rs6565922	T	C	0.38	0.078	0.016	1.54E-06
glycemic control	rs9384	T	G	0.38	-0.107	0.016	6.88E-11
glycemic control	rs10404726	T	C	0.47	-0.035	0.015	1.95E-02
glycemic control	rs58542926	T	C	0.08	0.139	0.029	2.27E-06
glycemic control	rs924150	C	A	0.39	-0.087	0.016	9.23E-08
glycemic control	rs4805881	C	A	0.67	-0.077	0.016	2.08E-06
glycemic control	rs429358	C	T	0.16	-0.142	0.021	4.30E-11
glycemic control	rs8107527	A	G	0.28	0.105	0.017	1.53E-09
glycemic control	rs9304665	A	T	0.77	0.103	0.018	2.01E-08
glycemic control	rs2115107	A	G	0.38	0.069	0.016	1.98E-05
glycemic control	rs116843064	A	G	0.02	-0.15	0.055	6.41E-03
glycemic control	rs34506349	A	G	0.04	-0.099	0.038	9.17E-03
glycemic control	rs79950062	C	T	0.13	-0.053	0.023	2.10E-02
glycemic control	rs9308614	G	A	0.15	-0.09	0.022	5.04E-05
glycemic control	rs6716394	A	G	0.54	-0.045	0.015	2.75E-03
glycemic control	rs4668483	G	A	0.68	-0.04	0.016	1.24E-02
glycemic control	rs10184004	T	C	0.41	-0.115	0.016	2.68E-12
glycemic control	rs13406280	T	C	0.49	-0.047	0.015	1.78E-03
glycemic control	rs72917531	A	C	0.19	-0.078	0.02	1.09E-04
glycemic control	rs36051007	T	C	0.32	-0.035	0.017	3.92E-02
glycemic control	rs67383253	C	T	0.37	-0.035	0.016	2.85E-02
glycemic control	rs6712905	C	T	0.26	0.048	0.018	7.67E-03
glycemic control	rs4482463	A	C	0.92	-0.063	0.029	2.96E-02
glycemic control	rs34329895	G	A	0.6	-0.063	0.016	9.39E-05

glycemic control	rs2943650	T	C	0.65	0.143	0.016	6.10E-18
glycemic control	rs13415288	C	T	0.34	0.059	0.016	2.48E-04
glycemic control	rs34339006	T	C	0.39	0.092	0.016	1.72E-08
glycemic control	rs1260326	C	T	0.61	0.156	0.016	6.16E-21
glycemic control	rs77165542	T	C	0.04	-0.155	0.042	2.46E-04
glycemic control	rs921069	G	A	0.58	-0.038	0.016	1.74E-02
glycemic control	rs76675804	C	T	0.1	-0.311	0.026	2.67E-30
glycemic control	rs10193538	T	G	0.61	0.072	0.016	8.71E-06
glycemic control	rs243018	G	C	0.45	0.088	0.016	6.64E-08
glycemic control	rs10188334	T	C	0.17	-0.087	0.02	1.69E-05
glycemic control	rs12185610	C	A	0.41	-0.063	0.016	9.39E-05
glycemic control	rs4671799	G	A	0.68	-0.036	0.016	2.43E-02
glycemic control	rs4832290	C	T	0.77	-0.053	0.018	3.29E-03
glycemic control	rs6137042	A	G	0.2	-0.05	0.019	8.50E-03
glycemic control	rs7274134	T	C	0.25	-0.062	0.018	6.08E-04
glycemic control	rs6059662	G	A	0.65	0.037	0.016	2.06E-02
glycemic control	rs2038457	G	A	0.81	0.041	0.02	4.00E-02
glycemic control	rs12625671	C	T	0.11	0.118	0.025	3.20E-06
glycemic control	rs6066138	A	G	0.28	-0.135	0.017	1.36E-14
glycemic control	rs6021276	C	T	0.64	-0.074	0.016	4.96E-06
glycemic control	rs865034	C	T	0.66	0.04	0.016	1.24E-02
glycemic control	rs4810145	C	T	0.52	0.068	0.015	7.51E-06
glycemic control	rs6011155	C	T	0.37	-0.074	0.016	4.96E-06
glycemic control	rs2240716	T	C	0.3	0.074	0.017	1.66E-05
glycemic control	rs56392746	A	G	0.09	-0.138	0.026	1.81E-07
glycemic control	rs75307421	A	G	0.02	0.151	0.061	1.32E-02
glycemic control	rs138771	G	A	0.81	-0.055	0.02	5.99E-03
glycemic control	rs1801645	T	C	0.74	-0.059	0.018	1.09E-03
glycemic control	rs17036126	T	C	0.13	0.127	0.023	5.92E-08
glycemic control	rs11708067	G	A	0.25	-0.262	0.018	1.55E-43
glycemic control	rs17036160	T	C	0.12	-0.088	0.024	2.69E-04
glycemic control	rs9873519	T	C	0.53	0.097	0.015	2.70E-10
glycemic control	rs667920	T	G	0.77	0.038	0.018	3.45E-02
glycemic control	rs9289556	T	C	0.73	-0.077	0.017	7.64E-06
glycemic control	rs56243018	C	A	0.05	-0.214	0.036	5.82E-09
glycemic control	rs28502438	C	T	0.43	-0.051	0.016	1.49E-03
glycemic control	rs7633673	A	G	0.41	-0.086	0.016	1.28E-07
glycemic control	rs11706810	C	T	0.48	-0.106	0.015	5.99E-12
glycemic control	rs13099581	T	C	0.14	-0.06	0.022	6.41E-03
glycemic control	rs8192675	C	T	0.29	-0.188	0.017	2.89E-26
glycemic control	rs6444036	T	G	0.16	0.041	0.021	5.05E-02
glycemic control	rs9859406	A	G	0.31	0.167	0.017	3.21E-21
glycemic control	rs2041965	T	C	0.34	-0.083	0.016	3.33E-07
glycemic control	rs6777684	G	A	0.61	0.134	0.016	5.25E-16

glycemic control	rs13094957	C	T	0.2	-0.131	0.019	1.84E-11
glycemic control	rs1470560	A	G	0.37	0.037	0.016	2.06E-02
glycemic control	rs2624847	T	G	0.74	-0.084	0.017	1.12E-06
glycemic control	rs13434089	C	T	0.12	-0.082	0.024	6.72E-04
glycemic control	rs9870517	C	A	0.4	-0.096	0.016	4.24E-09
glycemic control	rs1374915	C	T	0.42	-0.036	0.016	2.43E-02
glycemic control	rs1523766	G	A	0.5	-0.031	0.015	3.84E-02
glycemic control	rs978444	T	G	0.55	-0.057	0.015	1.61E-04
glycemic control	rs3872707	A	G	0.12	0.049	0.023	3.29E-02
glycemic control	rs7659468	G	T	0.49	-0.103	0.015	2.20E-11
glycemic control	rs11728350	G	A	0.13	0.11	0.023	2.39E-06
glycemic control	rs77141743	A	G	0.16	0.045	0.021	3.19E-02
glycemic control	rs730831	G	T	0.04	-0.123	0.041	2.75E-03
glycemic control	rs2604918	T	G	0.33	-0.063	0.016	9.39E-05
glycemic control	rs2125799	C	T	0.33	0.06	0.016	1.96E-04
glycemic control	rs28819812	A	C	0.32	-0.06	0.016	1.96E-04
glycemic control	rs4865436	G	C	0.29	0.05	0.018	5.51E-03
glycemic control	rs2169033	T	C	0.68	0.081	0.017	2.60E-06
glycemic control	rs55691245	A	G	0.14	-0.16	0.022	1.51E-12
glycemic control	rs7664347	C	T	0.64	-0.04	0.016	1.24E-02
glycemic control	rs10938398	A	G	0.43	0.04	0.016	1.24E-02
glycemic control	rs1996617	C	T	0.29	0.101	0.017	5.93E-09
glycemic control	rs114447556	T	C	0.08	0.08	0.029	5.84E-03
glycemic control	rs10937721	C	G	0.59	0.142	0.016	1.01E-17
glycemic control	rs73222806	G	C	0.05	0.098	0.035	5.15E-03
glycemic control	rs6835992	G	A	0.69	0.066	0.017	1.17E-04
glycemic control	rs993380	G	A	0.67	-0.059	0.016	2.48E-04
glycemic control	rs28408270	T	G	0.47	-0.05	0.015	9.01E-04
glycemic control	rs1961224	G	A	0.35	-0.065	0.016	5.67E-05
glycemic control	rs141146025	A	C	0.02	0.129	0.058	2.59E-02
glycemic control	rs75432112	A	G	0.05	0.195	0.036	1.03E-07
glycemic control	rs329118	T	C	0.42	0.041	0.016	1.04E-02
glycemic control	rs111686785	G	A	0.03	0.182	0.044	4.18E-05
glycemic control	rs72734782	G	A	0.21	0.066	0.019	5.47E-04
glycemic control	rs12514030	G	T	0.12	-0.103	0.023	9.60E-06
glycemic control	rs1650505	A	G	0.21	0.06	0.019	1.64E-03
glycemic control	rs4343858	A	G	0.23	-0.042	0.018	1.95E-02
glycemic control	rs138373837	T	C	0.02	0.101	0.05	4.30E-02
glycemic control	rs62366821	G	A	0.49	-0.055	0.015	2.69E-04
glycemic control	rs10067659	C	G	0.79	-0.081	0.019	2.45E-05
glycemic control	rs4865796	A	G	0.69	0.049	0.017	3.99E-03
glycemic control	rs464605	T	C	0.75	0.08	0.019	3.06E-05
glycemic control	rs34341	T	A	0.58	0.073	0.016	6.58E-06
glycemic control	rs7732130	A	G	0.68	-0.132	0.016	1.36E-15

glycemic control	rs6870983	T	C	0.21	-0.067	0.019	4.52E-04
glycemic control	rs34483452	A	C	0.14	0.077	0.023	8.56E-04
glycemic control	rs7752666	T	C	0.32	-0.035	0.017	3.92E-02
glycemic control	rs80196932	C	T	0.16	-0.064	0.021	2.36E-03
glycemic control	rs11759026	G	A	0.23	0.136	0.018	2.16E-13
glycemic control	rs2876354	T	C	0.47	-0.083	0.016	3.33E-07
glycemic control	rs7742292	C	T	0.4	0.041	0.016	1.04E-02
glycemic control	rs2982521	T	A	0.63	-0.11	0.016	2.09E-11
glycemic control	rs9390022	C	T	0.38	-0.042	0.016	8.66E-03
glycemic control	rs1538247	C	T	0.3	0.093	0.017	7.76E-08
glycemic control	rs2179168	A	G	0.8	0.046	0.019	1.54E-02
glycemic control	rs501470	G	T	0.47	-0.089	0.015	6.20E-09
glycemic control	rs4709746	T	C	0.13	-0.05	0.023	2.95E-02
glycemic control	rs7774074	A	C	0.21	0.039	0.019	3.98E-02
glycemic control	rs35261542	A	C	0.26	0.268	0.017	1.55E-50
glycemic control	rs3117189	G	A	0.85	0.281	0.021	3.05E-37
glycemic control	rs7748962	A	G	0.77	0.113	0.018	8.41E-10
glycemic control	rs9472139	C	G	0.29	0.065	0.017	1.47E-04
glycemic control	rs3798519	C	A	0.18	0.107	0.02	1.46E-07
glycemic control	rs9370243	T	G	0.08	0.079	0.028	4.82E-03
glycemic control	rs9449295	C	T	0.54	0.036	0.015	1.63E-02
glycemic control	rs9379084	A	G	0.12	-0.198	0.025	1.59E-14
glycemic control	rs187653072	C	T	0.03	0.134	0.044	2.38E-03
glycemic control	rs73184014	G	A	0.22	-0.053	0.019	5.32E-03
glycemic control	rs6976111	A	C	0.3	0.074	0.017	1.66E-05
glycemic control	rs13237518	A	C	0.41	0.048	0.016	2.75E-03
glycemic control	rs3996350	C	G	0.5	-0.086	0.015	1.89E-08
glycemic control	rs60251368	G	A	0.06	0.096	0.034	4.79E-03
glycemic control	rs4252505	G	A	0.06	0.07	0.031	2.38E-02
glycemic control	rs17168486	T	C	0.17	0.162	0.02	4.21E-15
glycemic control	rs4725959	G	A	0.22	0.042	0.019	2.68E-02
glycemic control	rs10228796	G	C	0.55	0.16	0.015	1.33E-24
glycemic control	rs6946660	C	T	0.35	-0.107	0.016	6.88E-11
glycemic control	rs2188848	G	A	0.2	-0.055	0.019	3.84E-03
glycemic control	rs860262	A	C	0.5	-0.158	0.015	4.73E-24
glycemic control	rs917195	T	C	0.23	-0.073	0.018	5.83E-05
glycemic control	rs730497	A	G	0.18	0.445	0.02	4.27E-97
glycemic control	rs73121277	C	T	0.28	0.084	0.017	1.12E-06
glycemic control	rs6975279	A	C	0.26	0.101	0.018	3.67E-08
glycemic control	rs6956980	C	T	0.53	0.083	0.015	5.56E-08
glycemic control	rs7834323	C	T	0.29	-0.074	0.017	1.66E-05
glycemic control	rs727582	G	A	0.34	-0.093	0.016	1.22E-08
glycemic control	rs13266634	T	C	0.31	-0.277	0.017	9.11E-54
glycemic control	rs12056338	T	G	0.42	0.05	0.016	1.83E-03

glycemic control	rs1561927	T	C	0.73	-0.048	0.017	4.79E-03
glycemic control	rs35753840	C	A	0.39	0.054	0.016	7.78E-04
glycemic control	rs13268508	T	C	0.38	0.087	0.016	9.23E-08
glycemic control	rs2953845	T	C	0.55	0.042	0.015	5.15E-03
glycemic control	rs6558173	T	G	0.35	0.039	0.016	1.47E-02
glycemic control	rs2725370	C	T	0.7	-0.049	0.017	3.99E-03
glycemic control	rs57735787	G	A	0.25	-0.042	0.018	1.95E-02
glycemic control	rs13262861	A	C	0.17	-0.121	0.021	1.61E-08
glycemic control	rs7813865	C	T	0.29	0.041	0.017	1.58E-02
glycemic control	rs10101067	C	G	0.08	0.092	0.029	1.56E-03
glycemic control	rs28792187	G	A	0.07	0.123	0.03	4.86E-05
glycemic control	rs1895874	A	G	0.5	0.048	0.015	1.42E-03
glycemic control	rs10808671	G	A	0.53	-0.073	0.015	1.61E-06
glycemic control	rs60384372	G	A	0.47	-0.056	0.015	2.09E-04
glycemic control	rs1567353	G	C	0.31	0.035	0.017	3.92E-02
glycemic control	rs10119430	A	G	0.79	-0.054	0.019	4.53E-03
glycemic control	rs1431819	G	A	0.7	0.038	0.017	2.52E-02
glycemic control	rs10818763	T	C	0.13	-0.108	0.023	3.58E-06
glycemic control	rs10739629	T	C	0.51	-0.036	0.015	1.63E-02
glycemic control	rs529565	C	T	0.32	0.164	0.017	1.52E-20
glycemic control	rs28642213	G	A	0.75	0.169	0.018	1.41E-19
glycemic control	rs12380322	G	A	0.39	0.051	0.016	1.49E-03
glycemic control	rs10965247	G	A	0.18	-0.302	0.02	1.27E-46
glycemic control	rs7018475	G	T	0.26	0.178	0.018	1.79E-21
glycemic control	rs11788619	T	A	0.03	-0.134	0.048	5.28E-03
glycemic control	rs2150854	T	G	0.33	0.072	0.016	8.71E-06
glycemic control	rs4237150	C	G	0.4	0.091	0.016	2.43E-08
glycemic control	rs67269808	G	A	0.06	-0.13	0.032	5.67E-05
glycemic control	rs2796441	A	G	0.42	-0.096	0.016	4.24E-09
glycemic control	rs7023781	T	C	0.27	0.058	0.017	6.83E-04
glycemic control	rs10993072	T	C	0.32	0.083	0.016	3.33E-07
glycemic control	rs28496034	G	C	0.33	-0.057	0.016	3.96E-04

Table S4. the main results of significant risk factors in univariable MR.

outcome	exposure	method	nsnp	pval	or	or_lci95	or_uci95
LS	PP	Simple median	76	0.032	1.05	1.00	1.10
LS	PP	Weighted median	76	0.338	1.02	0.97	1.08
LS	PP	MR Egger	76	0.888	1.01	0.89	1.14
LS	PP	IVW (fixed effects)	76	0.012	1.04	1.01	1.08
LS	DBP	Simple median	75	0.023	1.07	1.01	1.14
LS	DBP	Weighted median	75	0.021	1.07	1.01	1.14
LS	DBP	MR Egger	75	0.606	0.96	0.82	1.12
LS	DBP	IVW (fixed effects)	75	0.003	1.06	1.02	1.11
LS	SBP	Simple median	98	0.010	1.04	1.01	1.08
LS	SBP	Weighted median	98	0.002	1.05	1.02	1.08
LS	SBP	MR Egger	98	0.036	1.12	1.01	1.24
LS	SBP	IVW (fixed effects)	98	4.64E-07	1.06	1.03	1.08
LS	HDL cholesterol || id:ieu-b-109	Simple median	257	0.144	0.87	0.72	1.05
LS	HDL cholesterol || id:ieu-b-109	Weighted median	257	0.390	0.92	0.77	1.11
LS	HDL cholesterol || id:ieu-b-109	MR Egger	257	0.111	0.87	0.73	1.03
LS	HDL cholesterol || id:ieu-b-109	IVW (multiplicative random effects)	257	0.008	0.86	0.76	0.96
LS	apolipoprotein A-I || id:ieu-b-107	Simple median	226	0.217	0.89	0.73	1.07
LS	apolipoprotein A-I || id:ieu-b-107	Weighted median	226	0.554	0.95	0.79	1.13
LS	apolipoprotein A-I || id:ieu-b-107	MR Egger	226	0.352	0.91	0.76	1.10
LS	apolipoprotein A-I || id:ieu-b-107	IVW (multiplicative random effects)	226	0.034	0.88	0.78	0.99
LS	triglycerides || id:ieu-b-111	Simple median	234	0.001	1.45	1.18	1.80
LS	triglycerides || id:ieu-b-111	Weighted median	234	0.386	1.08	0.91	1.29
LS	triglycerides || id:ieu-b-111	MR Egger	234	0.861	1.02	0.85	1.21
LS	triglycerides || id:ieu-b-111	IVW (multiplicative random effects)	234	0.028	1.14	1.01	1.29
LS	apolipoprotein B || id:ieu-b-108	Simple median	142	0.190	1.14	0.94	1.39
LS	apolipoprotein B || id:ieu-b-108	Weighted median	142	0.546	1.06	0.88	1.26
LS	apolipoprotein B || id:ieu-b-108	MR Egger	142	0.377	1.09	0.90	1.31
LS	apolipoprotein B || id:ieu-b-108	IVW (multiplicative random effects)	142	0.030	1.15	1.01	1.31
LS	Type 2 diabetes	Simple median	113	0.001	1.16	1.06	1.26
LS	Type 2 diabetes	Weighted median	113	0.007	1.15	1.04	1.28
LS	Type 2 diabetes	MR Egger	113	0.190	1.09	0.96	1.24
LS	Type 2 diabetes	IVW (fixed effects)	113	1.02E-04	1.12	1.06	1.18
LS	Height	Simple median	290	0.204	0.92	0.81	1.05
LS	Height	Weighted median	290	0.278	0.93	0.81	1.06

LS	Height	MR Egger	290	0.034	0.76	0.59		0.98
LS	Height	IVW (multiplicative random effects)	290	0.011	0.88	0.81		0.97
LS	Education	Simple median	30	0.075	0.59	0.33		1.05
LS	Education	Weighted median	30	0.084	0.58	0.32		1.07
LS	Education	MR Egger	30	0.568	0.46	0.03		6.56
LS	Education	IVW (fixed effects)	30	0.006	0.55	0.36		0.84
LS	Fasting proinsulin	Simple median	9	0.030	2.19	1.08		4.45
LS	Fasting proinsulin	Weighted median	9	0.024	1.80	1.08		3.00
LS	Fasting proinsulin	MR Egger	9	0.517	1.29	0.62		2.66
LS	Fasting proinsulin	IVW (fixed effects)	9	0.012	1.54	1.10		2.15
LS	Fibrinogen	Simple median	33	0.245	2.13	0.59		7.65
LS	Fibrinogen	Weighted median	33	0.153	2.36	0.73		7.67
LS	Fibrinogen	MR Egger	33	0.171	5.13	0.52		50.58
LS	Fibrinogen	IVW (fixed effects)	33	0.020	2.52	1.16		5.47
LS	Atrial fibrillation	Simple median	74	0.357	0.95	0.85		1.06
LS	Atrial fibrillation	Weighted median	74	0.288	0.95	0.85		1.05
LS	Atrial fibrillation	MR Egger	74	0.111	0.90	0.79		1.02
LS	Atrial fibrillation	IVW (fixed effects)	74	0.010	0.92	0.86		0.98

Table S5. Multivariable MR estimates for blood pressure and lipids.

Outcome	Method	SNP N	Exposure	BETA	SE	p-value
ls	IVW	255	DBP	0.082	0.235	0.729
			SBP	-0.024	0.239	0.919
			PP	0.064	0.241	0.789
	MR-Egger	255	DBP	0.08	0.237	0.735
			SBP	-0.025	0.239	0.918
			PP	0.065	0.241	0.788
ls	IVW	384	ApoB	0.171	0.296	0.564
			LDL	-0.136	0.315	0.665
			TG	0.196	0.071	0.005*
	MR-Egger	384	ApoB	0.14	0.295	0.636
			LDL	-0.192	0.316	0.543
			TG	0.151	0.075	0.045*
	MR-Lasso	357	ApoB	-0.006	0.261	0.98
			LDL	0.035	0.28	0.902
			TG	0.261	0.063	0.000*
ls	IVW	435	ApoAI	0.023	0.187	0.900
			HDL	-0.201	0.178	0.26
	MR-Egger	435	ApoAI	0.065	0.194	0.738
			HDL	-0.187	0.179	0.296
	MR-Lasso	408	ApoAI	-0.046	0.167	0.783
			HDL	-0.149	0.161	0.354

Table S6. MR-Egger intercept and Q test result in the study.

outcome	exposure	IVW-Q test		Egger regression (intercept)		
		Q-statistic	Q_pval	egger_intercept	se	pval
**univariable MR**						
lacunar stroke	PP	90.6	0.106	0.005	0.010	0.589
lacunar stroke	DBP	90.0	0.099	0.014	0.011	0.178
lacunar stroke	SBP	106.6	0.238	-0.013	0.012	0.256
lacunar stroke	HDL	326.9	0.002	0.000	0.003	0.873
lacunar stroke	apolipoprotein A-I	289.5	0.002	-0.001	0.003	0.614
lacunar stroke	triglycerides	313.7	0.000	0.005	0.003	0.073
lacunar stroke	apolipoprotein B	195.6	0.002	0.003	0.004	0.394
lacunar stroke	Type 2 diabetes	291.8	0.142	0.004	0.003	0.153
lacunar stroke	Height	391.2	0.000	0.005	0.004	0.209
lacunar stroke	Education	20.0	0.894	0.003	0.024	0.891
lacunar stroke	Fasting proinsulin	11.2	0.190	0.009	0.015	0.578
lacunar stroke	Fibrinogen	63.6	0.001	-0.008	0.011	0.491
lacunar stroke	Atrial fibrillation	108.0	0.083	0.004	0.005	0.405
**multivariable MR**						
lacunar stroke	blood pressure: PP SBP PP	287.6	0.061	0.000	0.003	0.951
lacunar stroke	lipids: ApoB LDL TG	524.0	0.000	0.003	0.002	0.085
lacunar stroke	lipids: apolipoprotein A-I HDL	550.2	1.00E-04	-0.002	0.002	0.434
